# Random Forest Algorithm for the Mechanical Strength Prediction of Green Cement-Based Materials Incorporating Waste Materials Under Fire Condition

**DOI:** 10.3390/ma18051025

**Published:** 2025-02-26

**Authors:** Lei Zhang, Ruipeng Qiu, Jiabin Xie, Xianglong Liu, Qiang Fu, Yanli Li

**Affiliations:** 1 School of Water Conservancy and Civil Engineering, Northeast Agricultural University, Harbin 150030, China; 2 Department of Applied Mathematics, The Hong Kong Polytechnic University, Hung Hom, Kowloon, Hong Kong, China; 3 The School of Civil Engineering, Harbin University, Harbin 150030, China; 4 School of Infrastructure Engineering, Nanchang University, Nanchang 330031, China; 5 Architectural Design and Research Institute of HIT, Harbin Institute of Technology, Harbin 150090, China

**Keywords:** cement-based materials, waste material utilization, high temperature treatment, algorithm prediction

## Abstract

High temperature treatment is a typical detrimental situation that may significantly influence the compressive strength of cement-based materials. It was reported that the incorporation of common waste materials as supplementary cementitious materials (SCMs) can improve high temperature resistance. In this work, fly ash (FA), granulated blast-furnace slag (GGBFS), and silica fume (SF) were used as SCMs to replace cement to produce green cement-based materials. The mechanical strengths of the samples being subjected to various elevated temperatures were measured and analyzed with different SCMs contents. Results showed that when the high temperature was above 500 °C, it caused significant loss of strength, and the use of SCMs can improve the high temperature resistance of the cement-based materials with higher residual strength, especially for the GGBFS and SF blended samples. Moreover, the random forest regression algorithm was used to predict the compressive strength for the cement-based material incorporating various waste materials, and exhibited high accuracy. This work presents a comprehensive study on the regularity of changes of mechanical strength and provides a specific algorithm for the precise prediction of this occurrence, which is helpful to understand and predict the influence of high temperature treatment on green cement-based materials with various waste materials.

## 1. Introduction

Cement-based materials are some of the most popular nonorganic materials worldwide, due to being low price, easy to produce, and possessing good mechanical properties [1,2]. However, cement-based materials will meet a significant performance degradation during the fire conditions, causing a significant loss of strength and service performance, resulting in a severe risk to human safety and economic loss [3,4,5]. In general, the degraded performance of cement-based materials under fire conditions is related to the water vapor inside the structure generating high internal pressure; the expansion process weakens the pore structure of the structure [6,7]. Also, the structure will experience a series of phase decomposition process, which are also detrimental to the service life of cement-based materials [8,9]. In this context, the problem of how to mitigate fire-induced performance degradation in cement-based materials is pressing and muse be solved. In addition, more accurate prediction of the performance of the materials and the state of structures under fire conditions is needed.

Thus far, scholars have produced many studies focusing on mitigating the fire induced damage of cement-based materials; among which, the use of supplementary cementitious materials (SCMs) is noted as a useful approach [10,11,12]. Specifically, the inclusion of SCMs can improve the thermal stability of concrete and enhance the fire resistance of the samples, showcasing higher compressive strength after being subjected to elevated temperatures, as SCMs exhibit a pozzolanic reaction under high temperatures of 200–500 °C, which can resist the damage caused by high temperatures [13,14,15]. Also, the inclusion of SCMs can mitigate the spalling inside the sample; especially for samples with high strength, their dense pore structure always tends to show high pore pressure, resulting in the spalling of the sample. When the SCMs are added in the sample, the spalling phenomenon can be effectively limited. To summarize, the incorporation of SCMs in cement-based materials provides benefits in terms of high temperature resistance, with high thermal stability, and improved high temperature resistance [16,17,18]. Moreover, the use of SCMs as a substitute for cement is also beneficial for reducing CO_2_ emissions, which is in accordance with the environmental protection policy [19,20,21]. Among the SCMs, fly ash (FA), granulated blast-furnace slag (GGBFS), and silica fume (SF) are popular, which were proven to effectively improve the performance of cement-based materials with refined pore structures and enhanced hydration degrees [22,23,24].

Based on the discussion above, it can be concluded that the inclusion of SCMs in cement-based materials can relieve high temperature-caused performance degradation. At this time, systemic research about the specific effect of various SCMs at different contents on resisting fire-induced damage is in great demand. Also, how to accurately predict the compressive strength of SCMs blended cement-based materials is important, because it is time wasting and costly to conduct large-scale experiments. Under this circumstance, the utilization of the machine learning (ML) method is an effective way to solve the problem [25,26,27]. ML can offer various approaches to solve the problem by leveraging data from previous tests and historical data on cement-based materials treated by various conditions [28,29,30]. And, if enough data can be provided for the ML, the strength performance of cement-based materials containing various SCMs after being subjected to high temperatures can be predicted with high accuracy. To build on basic research on the performance of cement-based materials under fire conditions and methods to predict this, traditional mineral materials can be included to obtain more universal conclusions.

In this work, three common mineral materials, FA, GGBFS, and SF, were used as the SCMs to replace cement, and the contents of the SCMs were varied within the range of 0–40%. The compressive strengths of blended cement-based materials were measured after being subjected to various high temperatures ranging from 250 °C to 1000 °C, aiming to disclose the macroscopic performance evolution of blended cement-based materials after high temperature conditions. Furthermore, to better predict the performance of high temperature treated cement-based materials, the random forest regression (RFR) method was used as the machine learning method, and the model performance of RFR method was further evaluated.

## 2. Materials and Methods

### 2.1. Raw Materials

Ordinary Portlandite cement (produced by Fushun Cement Corporation, Fushun, China) with the strength of 42.5 MPa was used as the main cementitious material; FA was purchased from Harbin Xingre fly ash development company (Harbin, China). GGBFS and silica fume were also used as the cementitious materials (produced by Tangshan commercially available company, Tangshan, China). The chemical compositions of raw powder materials are listed in Table 1. The standard sand produced by Aisio Ltd., Sutton, UK was used as the fine aggregate to prepare cement mortar; the SiO_2_ content in the sand is greater than 96%, and the sand is classified as natural quartz sea sand. A polycarboxylic-acid based water reducer with a solid content of 30% was used.

### 2.2. Sample Preparation Method

The cement was replaced by FA, GGBFS, and SF with mass ratios of 10%, 20%, 30%, and 40%, respectively. Only one waste material at a time was used to replace cement. The water-to-binder ratio was kept at 0.35, and the sand-to-binder ratio was set to 1.5. The mixing process is detailed as follows. All the powder materials and the fine aggregate were mixed together in a stirring pot and stirred for 3 min, then the solution of water and polycarboxylic acid-based water reducer was added, and the mixture was further stirred for 3 min. The newly prepared mixture was put in a mold with the size of 40 mm × 40 mm × 40 mm. Then, the mixtures were put in the curing room with the temperature of 20 ± 2 °C and relative humidity of >95%. The curing duration of the samples was 28 days; then, the samples were taken out and dried for 3 days at a temperature of 60 °C to exclude the water inside the samples.

### 2.3. High Temperature Treatment

**Figure 1:**
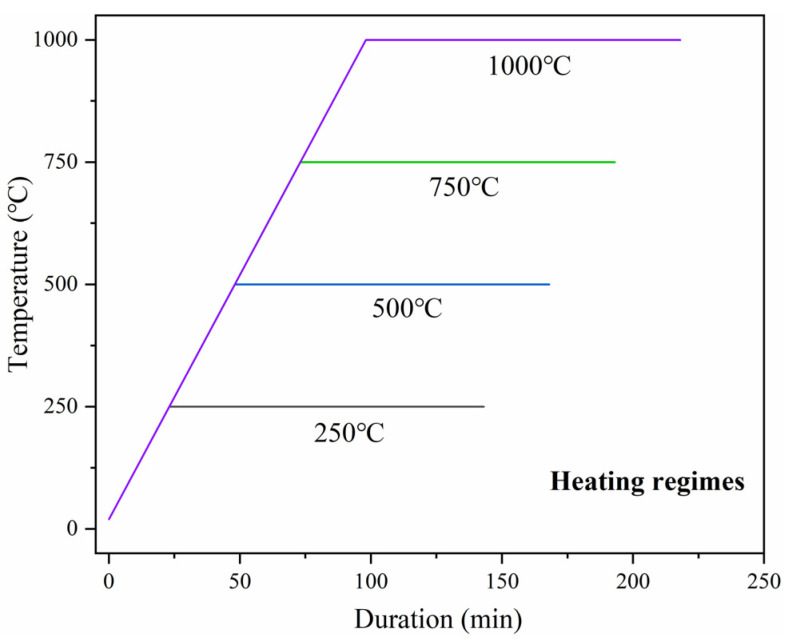
Heating procedure diagram.

When the samples were dried, the high temperature treatment was conducted as shown in Figure 1. Four high temperature steps of 250 °C, 500 °C, 750 °C, and 1000 °C were chosen for the test. The heating rate during the temperature rising process was set to 10 °C/min, and the holding time at the peak temperature was set to 2 h. Then, the samples were cooled naturally.

### 2.4. Mechanical Strength Test

The compressive strengths of the samples were first tested after 28 days of curing and were also measured after the high temperature treatment. During the test process, the force loading rate was kept at 2.4 kN/s and three samples were measured in each batch and the average value was taken as the representative.

### 2.5. Algorithm Impementation

**Figure 2:**
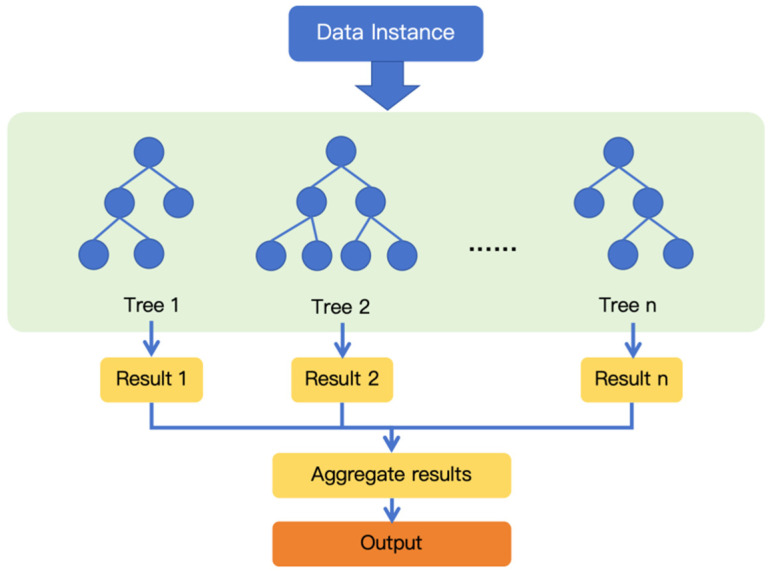
Illustration of the procedure of RFR method.

Random forest regression (RFR) is a classic ensemble method used for predicting the numerical values in different cases [31,32] and this method is utilized in this work to predict the strength development of cement-based materials after high temperature treatment. Generally, RFR can improve the generalization performance by constructing an ensemble of decision trees that are decorrelated from each other [33,34,35]. Given a training dataset D consisting of m instances and n attributes, the general procedure of training a RFR is illustrated in Figure 2, and is described as follows [36,37]:

(1) Bootstrap sampling: Construct a dataset $D_{i}$ by randomly sampling m instances (with replacement) from the original dataset D.
(2) Building an individual decision tree: Train a decision tree $T_{i}$ by spliting the data recursively at each node based on dataset $D_{i}$. The splitting is repeated until a stopping condition is met (e.g., Squared Error). At every internal node of $T_{i}$, a subset of attributes is randomly selected, and the best attribute form the subset is selected, which achieves the maximum reduction in error to split the data. In general, the number of attributes in the subset is smaller than the total number of attributes in the dataset D.
(3) Repeat steps (1) and (2) until all decision trees are trained.
(4) Aggregating predictions: Once all decision trees have been constructed, the average prediction of trees is used as the final prediction of the random forest.

## 3. Results and Discussion

### 3.1. Mechanical Strength Development of Cement Mortar Incorporating FA

**Figure 3:**
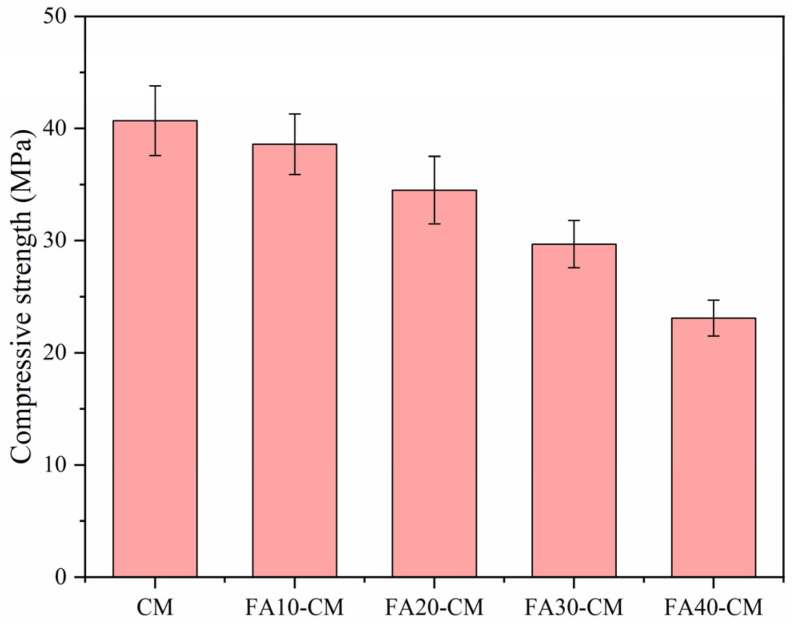
Compressive strengths of FA blended samples before high temperature treatment.

Figure 3 depicts the compressive strengths of the cement mortar samples including various contents of FA. It can be seen that the compressive strengths showed a decreasing trend with the increasing FA contents, which was consistent with the published literature suggesting FA may induce the strength declination at early curing ages because of the low activity characteristic [38,39]. To be specific, for the pure cement mortar sample, the compressive strength at 28 days was above 40 MPa, which became around 25 MPa when the FA content was 40%, meeting a comparable reduction.

**Figure 4:**
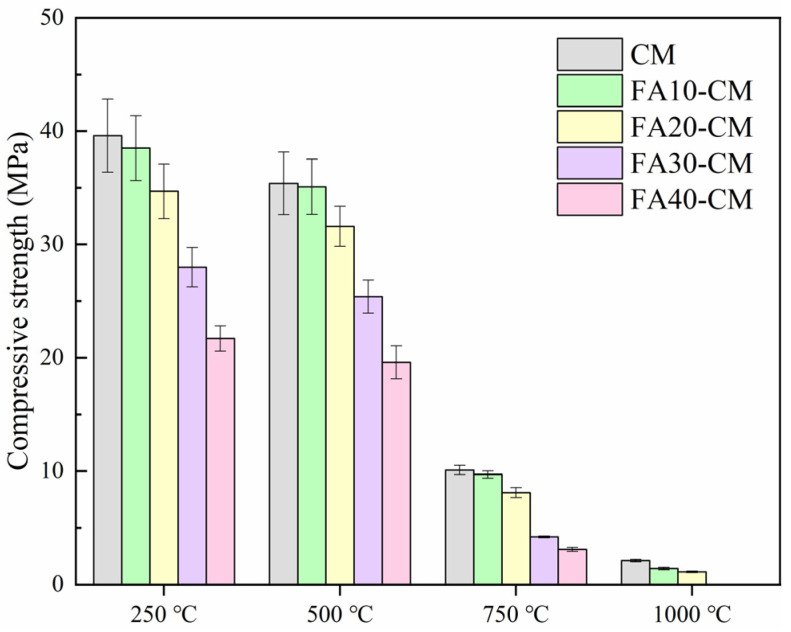
Compressive strengths of FA blended samples after being subjected to high temperature treatment.

The mechanical strengths of FA blended samples after being subjected to high temperature treatment are shown in Figure 4. The high temperature treatment was detrimental to the performance of cement mortar, and this can be verified by the significantly reduced strength. It should note that when the temperatures were lower than 500 °C, the strength reduction for FA blended samples was not obvious. However, remarkable loss of strength can be found when the temperatures were increased. To be specific, the compressive strength of the samples met the reduction smaller than 5% when the temperature was 250 °C; when the temperature increased to 500 °C, the strength losses were still concentrated in 15%. Oppositely, the compressive strengths of the samples met significant reduction higher than 70% when the temperature was 750 °C. Compared to the pure cement mortar, FA exhibited the ability on refining the strength reduction trend of the samples, especially when the FA content was lower than 20%. This was because the compressive strength for the samples was high enough to resist the high temperature and the pozzolanic reaction between FA and hydration products under high temperatures and could compensate for part of the high temperature attack. However, when the FA contents were higher than 20%, the compressive strengths could not even be tested at 1000 °C, because the samples were totally damaged.

### 3.2. Mechanical Strength Development of Cement Mortar Containing GGBFS

**Figure 5:**
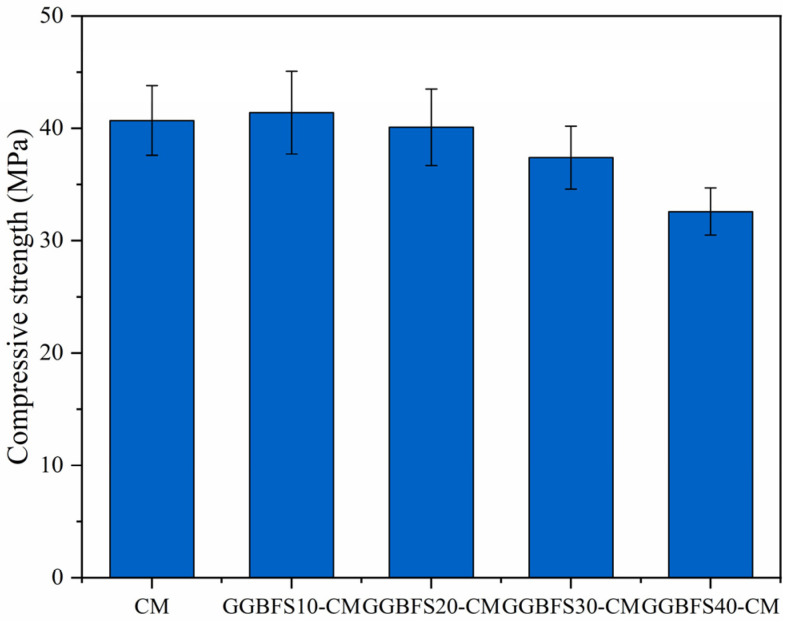
Compressive strengths of cement mortar containing various GGBFS contents.

Different from the effect of FA, the inclusion of GGBFS partly increased the 28-day compressive strengths of the samples, as depicted in Figure 5. Specifically, the compressive strengths of GGBFS10-CM and GGBFS20-CM samples were close to that of the pure mortar sample, and the strength of GGBFS10-CM was even higher than the reference group, indicating a higher reaction activity of GGBFS than FA in the matrix.

**Figure 6:**
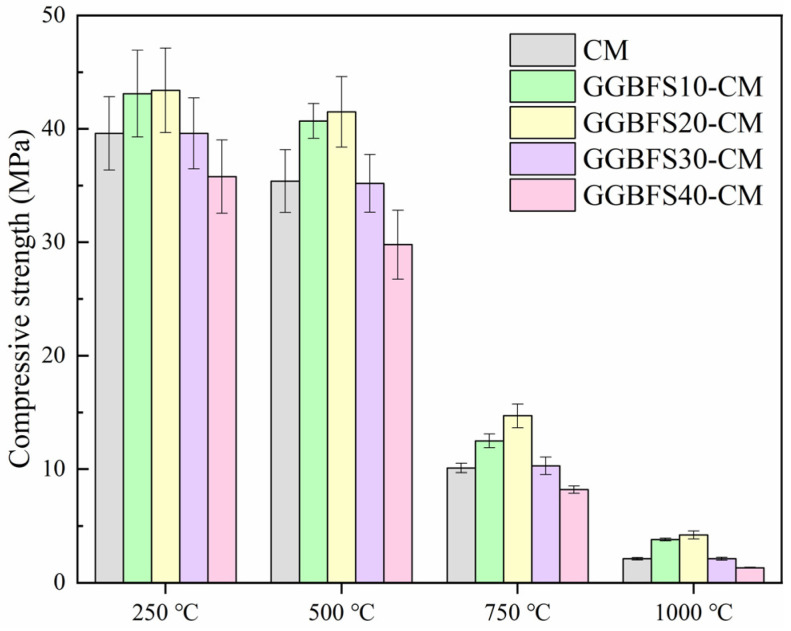
Compressive strengths of GGBFS-CM after being subjected to high temperature treatment.

As for the high temperature resistance of GGBFS blended samples, the mechanical strengths are depicted in Figure 6. It can be found that the strength-changing regularity for GGBFS blended samples was different from that of FA blended samples. A more significant pozzolanic reaction can be found in GGBFS-CM samples; the compressive strengths of GGBFS10-CM and GGBFS20-CM were much higher than that of the pure mortar sample because the pozzolanic reaction between GGBFS and Ca(OH)_2_ promoted the strength improvement [40,41,42]. This can also explain the phenomenon that occurred when the high temperature were 250 °C and 500 °C; the compressive strengths for GGBFS-CM samples with 10% and 20% GGBFS addition were even higher than that of the pure sample without being subjected to high temperatures. A suitable high temperature condition may stimulate the pozzolanic reaction inside the sample, resulting in improved compressive strengths.

### 3.3. Mechanical Strength Development of Cement Mortar Containing SF

**Figure 7:**
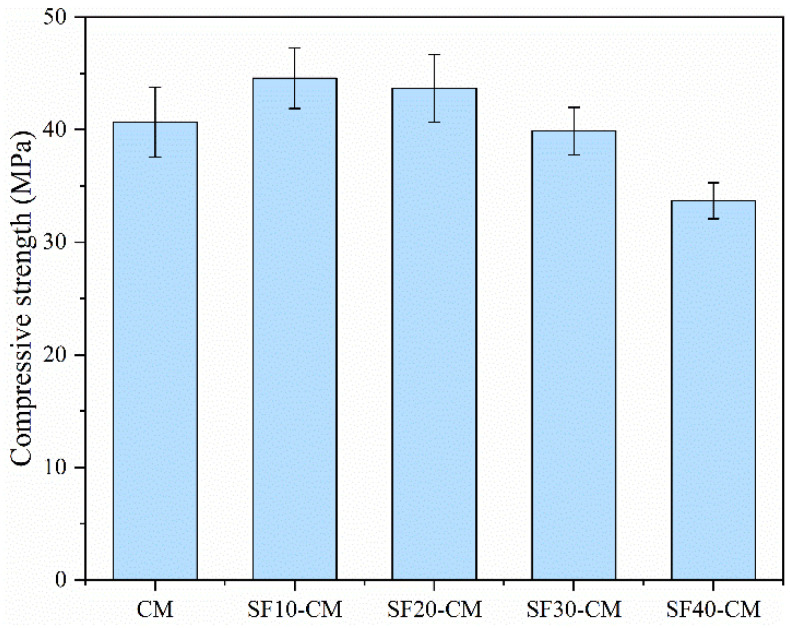
Compressive strengths of cement mortar containing various SF contents.

As a typical SCM with high activity, the inclusion of SF significantly enlarged the compressive strength of the samples, as depicted in Figure 7. The compressive strengths were 9.6% and 7.4% improved by 10% and 20% SF replacement, with the values of 44.6 MPa and 43.7 MPa, and the strength reduction ratio was also much decreased with higher SF contents compared to those of FA and GGBFS. This increased strength can be attributed to two reasons. On the one hand, the smaller particle size of SF made this material exhibit a specific filling effect. On the other hand, the high activity of SF guaranteed sufficient reaction in the sample [43,44].

**Figure 8:**
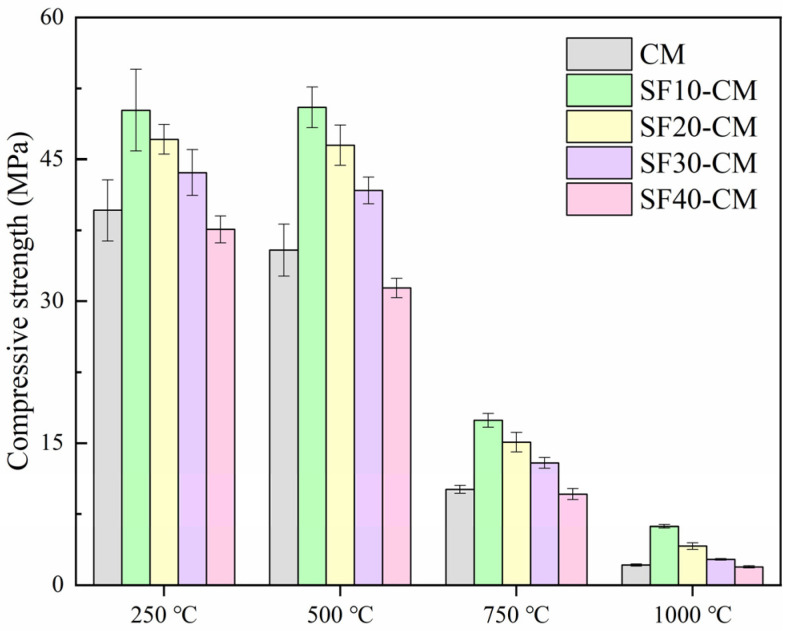
Compressive strengths of SF-CM after being subjected to high temperature treatment.

The compressive strengths of SF blended samples after being subjected to high temperature treatment are depicted in Figure 8. It can be found that the pozzolanic reaction of SF was significantly improved compared to FA and GGBFS. After high temperature treatment at 250 °C, the compressive strengths of the samples reached 50.2 MPa, 47.1 MPa, 43.6 MPa, and 37.6 MPa, respectively, with the SF contents increasing from 10% to 40%. These values became 35.4 MPa, 50.5 MPa, 46.5 MPa, 41.7 MPa, and 31.4 MPa for the 500 ℃-treated samples as the SF contents varied from 0 to 40%. It can be found that the strengths of the samples were still higher than the untreated pure cement mortar even after treatment at 500 °C with a high SF content of 30%, indicating the more intense pozzolanic reaction between SF and Ca(OH)_2_ under high temperatures.

### 3.4. Discussion over the Strengh Development of the Cement Morater After High Temperature Treatment

**Figure 9:**
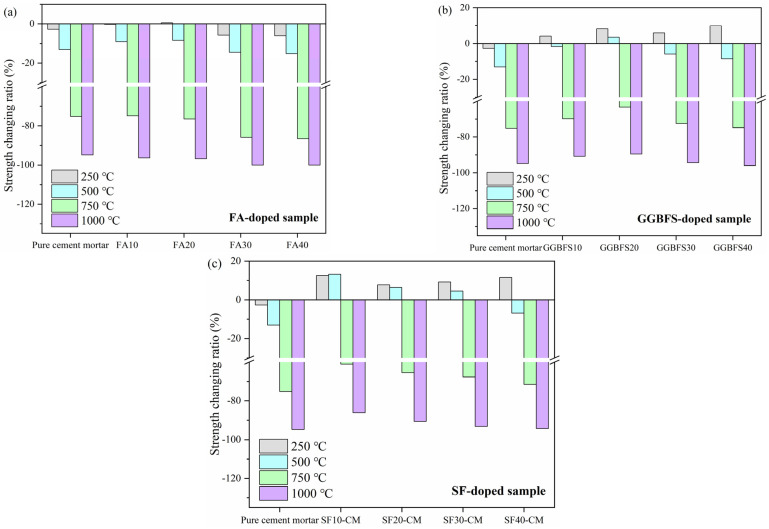
Strength-changing ratios of cement mortar incorporating different waste materials after being subjected to high temperature treatment. (**a**) FA-doped sample (**b**) GGBFS-doped sample (**c**) SF-doped sample.

Figure 9 exhibits the strength-changing ratios of the sample at different high temperatures. The strength-changing ratio reflected the ability of the sample to resist the damage induced by the high temperature. It can be observed that the FA blended samples exhibited a negative strength-changing trend; the strength losses were more significant in FA blended samples compared to the pure cement mortar when the temperature was higher than 500 °C. This phenomenon indicated that as a typical SCM, FA-CM showed a poor ability to maintain the strength of the sample, and the structure was totally destroyed in FA30-CM and FA40-CM samples at 1000 °C because the strength loss reached 100%. Conversely, as depicted in Figure 9b, GGBFS exhibited a positive influence on the strength-changing trend for the high temperature treated samples. To be specific, when the temperature was 250 °C, all the GGBFS blended samples showed a positive strength-changing trend, indicating that the hydration reaction in the samples was improved at this temperature. As for the sample doped with 20% GGBFS, the strength was also increased when the temperature was 500 °C, while the strengths were negatively changed in other samples. Under higher temperatures, the strength-changing ratios for the samples exhibited an increasing then decreasing trend, and the optimal content was found to be 20%, as the strength-decreasing ratios were always lower in this sample than others. As demonstrated in Section 3.3, SF was proven to effectively improve the compressive strength of high temperature treated sample. In this section, the benefits of SF on refining the performance of the samples are clearly noticeable. It was found that the strength-changing ratios were all positive when the temperatures were 250 °C and 500 °C, except SF40-CM; this showed the high pozzolanic activity of SF under a suitable high temperature. Therefore, the optimal content of SF was 10%, with the lowest strength-decreasing ratio in the sample.

To summarize, the kind of SCMs, the SCM content, and the heating temperature all influenced the compressive strength of cement mortar and understanding how to predict the compressive strength of the sample after being subjected to high temperatures is of vital importance to better understand the actual behavior of the samples under fire conditions. In the following section, RFR was conducted to predict the strength of cement mortar under different temperatures.

### 3.5. Algorithm Implementation of RFR to Predict the Compressive Strength of Blended Samples

#### 3.5.1. The Establishment of RFR Model

The RFR model was trained and tested based on experimental data, which contained 75 samples and 4 variables. Among them, “Temperature”, “Content”, and “SCMs” were independent variables and “Strength” was the dependent variable. The examples of data instance are shown in Table 2. In order to train and test the model, the dataset was randomly divided into training set and testing set with a ratio of 0.8/0.2. Since “SCMs” was a categorical attribute, it was encoded into computable form with the one-hot encoding method. To be specific, one-hot encoding is a technique that converts categorical variables into a binary format by creating a new column for each unique category, with ‘1’ indicating the presence of that category and ‘0’ indicating its absence. However, to avoid the so called “perfect multicollinearity” (also known as dummy variable trap), it is common practice to drop one of the encoded columns. For example, if there are three categories, ‘A’, ‘B’, and ‘C’, you would create three columns (‘A’, ‘B’, and ‘C’), but typically drop one column (e.g., ‘C’) to prevent a linear dependency among the columns, ensuring model stability and interpretability.

#### 3.5.2. Model Training and Testing

**Figure 10:**
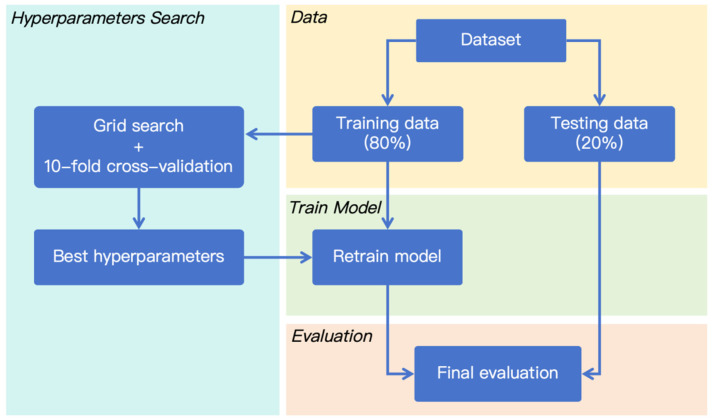
Procedure of model establishment.

The program of training and testing the RFR model was developed in a Python environment. Figure 10 shows the procedure of the model establishment. Since hyperparameters depict a significant impact on RFR model performance, hyperparameter tuning was conducted by grid search method with 10-fold cross-validation on the training set. The optimal hyperparameter settings are shown in Table 3. After hyperparameter tuning, a final model was trained on the whole training set with the optimal hyperparameters.

After training the RFR model, we evaluated its performance on the testing set based on $R^{2}$ and RMSE. R-Square ($R^{2}$) represents the proportion of variance in the dependent variable that can be explained by the independent variables, indicating the goodness of fit of a model. It is calculated as

$$R^{2}=1-\frac{\sum_{i=1}^{N}{\left(y_{i}^{*}-y_{i}\right)}^{2}}{\sum_{i=1}^{N}{\left(\bar{y}-y_{i}\right)}^{2}}$$

Root mean square error (RMSE) measures the error between predicted and real values. It is determined by

$$RMSE=\sqrt{\frac{1}{N}\sum_{i=1}^{N}{\left(y_{i}^{*}-y_{i}\right)}^{2}}$$

where $y_{i}^{*}$ and $y_{i}$ stand for the predicted and real strength value of the ith data sample, respectively. N represents the number of data samples.

**Figure 11:**
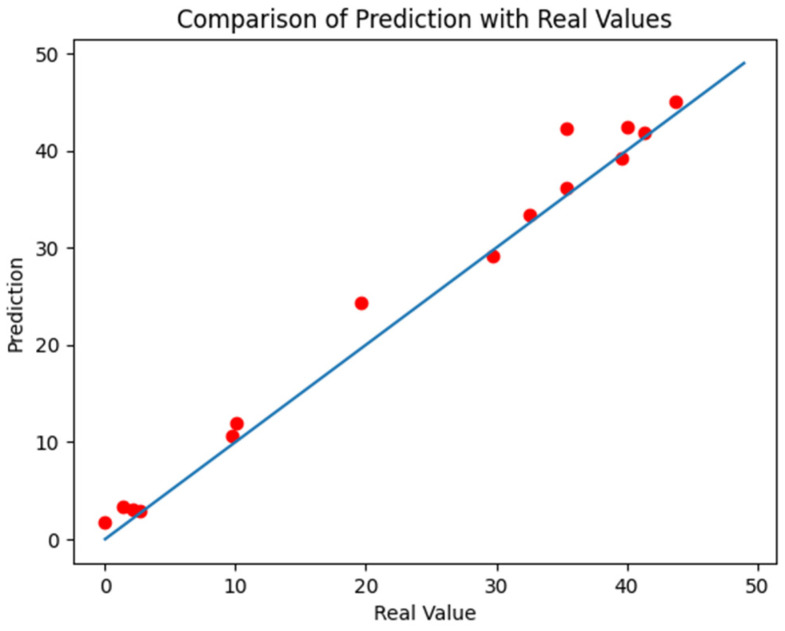
Comparison between real and predicted values.

The RFR model was evaluated on the testing set, achieving an $R^{2}$ of 0.98 and an RMSE of 2.46, which showed its good performance. To visually demonstrate the model’s performance, Figure 11 was plotted based on the real and predicted strength values of the testing samples. The *x*-axis represents the real values, while the *y*-axis represents the predicted values. Almost all data points in the figure are located close to the diagonal line, which demonstrates the model’s strong predictive capability.

**Figure 12:**
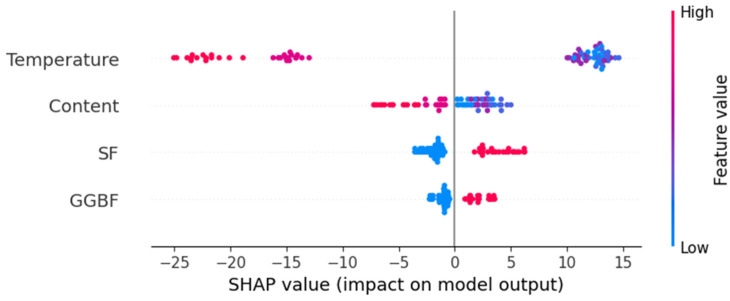
Summary graph of SHAP analysis.

**Figure 13:**
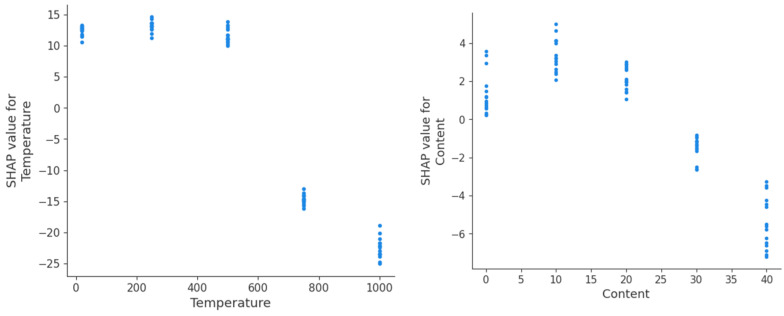

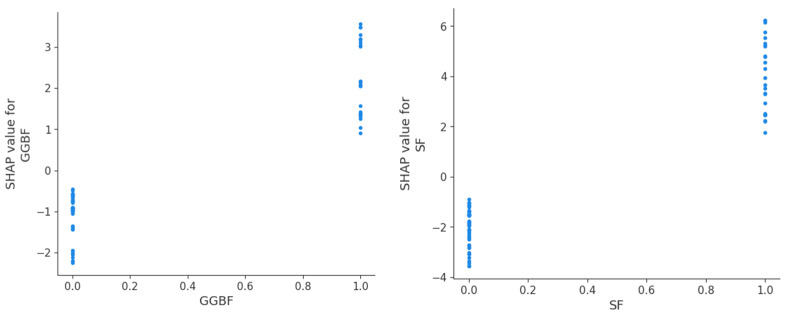
SHAP values of different attributes on strength.

To better understand the impact of different attributes on the dependent variable “strength”, this study implements SHapley Additive exPlanations (SHAP) to analyze the contribution of each attribute to the predictions of the RFR model. Figure 12 is a summary graph of SHAP analysis. Figure 13 depicts the SHAP values of each attribute. Since column “SCMs” was encoded into two columns [“GGBF”, “SF”] by the one-hot encoding method, SHAP analysis was performed for “GGBF” and “SF” instead of “SCMs”. A positive SHAP value indicates that the feature value has a positive influence on the strength, while a negative SHAP value indicates the opposite. The larger the absolute value of the SHAP value, the stronger the impact.

## 4. Conclusions

In this work, three common SCMs (FA, GGBFS and SF) were used in cement-based materials, aiming to relieve the damage inside the samples caused by high temperatures. Mechanical strength test results showed that the inclusion of FA exhibited few influences on the strength, and FA blended samples suffered from the most significant strength degradation under high temperature, which was related to the low hydration activity of FA. The inclusion of GGBFS depicted opposite trend that GGBFS improved the strengths of cement mortar, with a much-improved high temperature resistance ability, due to the higher pozzolanic reaction of GGBFS in cement mortar under high temperatures. Moreover, SF endowed the highest strength-increasing ratios for the samples and significantly lessened damage after exposure to fire conditions. When the high temperatures were lower than 500 °C, the inclusion of SF could effectively improve the compressive strengths for cement mortar, because the reactivity of SF was much higher than other two materials. The high strength provided the foundation for the sample to resist the high temperature damage. In addition, the random forest regression (RFR) method was used to establish the model for predicting the compressive strength of blended cement-based materials after high temperature damage, the attributes of different variables including SCMs type, treatment temperature, and SCM content were all considered and clarified, the model showed good performance and provided a reliable prediction model for the compressive strength of fire treated cement-based materials.

## Figures and Tables

**Table 1:** Chemical compositions of raw materials (wt%).

	SiO_2_	CaO	Al_2_O_3_	Fe_2_O_3_	MgO
Cement	22.13	63.47	6.32	3.56	1.77
FA	48.41	8.45	17.88	3.96	0.89
GGBFS	35.23	27.41	15.38	13.12	7.79
SF	95.42	0.38	0.41	0.32	0.25

**Table 2:** Examples of data instances.

SCMs	Temperature	Content	Strength
SF	250	10	50.2
SF	750	20	15.1
FA	20	10	38.6
GGBFS	1000	40	1.3

**Table 3:** Hyperparameter search and settings.

Hyperparameter	Description	Search Space	Optimal Value
max_depth	The maximum depth of the tree.	[2, 3, 4, …, 9]	8
min_samples_leaf	The minimum number of samples required to be at a leaf node.	[1, 2, 3, …, 9]	1
n_estimators	The number of trees in the forest.	[10, 20, 30, …, 100]	60

## Data Availability

The raw data supporting the conclusions of this article will be made available by the authors on request. The data are not publicly available due to the privacy issue.
